# Nursing Students’ Perceptions of Menstrual Distress during Clinical Practice: A Q-Methodology Study

**DOI:** 10.3390/ijerph18063160

**Published:** 2021-03-18

**Authors:** Ya-Lin Fu, Chia-Ling Yang, Shu-Chuan Yu, Yun-Hsuan Lin, Hsiao-Pei Hsu, Chiu-Mieh Huang

**Affiliations:** 1 Department of Nursing, College of Nursing, National Yang Ming Chiao Tung University, Taipei 112, Taiwan; s099@mail.mkc.edu.tw (Y.-L.F.); sandylucy616@ym.edu.tw (H.-P.H.); 2 Department of Nursing, School of Nursing, National Yang-Ming University, Taipei 112, Taiwan; 3 Department of Nursing, MacKay Junior College of Medicine, Nursing and Management, Taipei 112, Taiwan; s098@mail.mkc.edu.tw; 4 Yonghe Cardinal Tien Hospital, New Taipei 231, Taiwan; vivian916wu@gmail.com; 5 Department of Nursing, Ching Kuo Institute of Management and Health, Keelung 203, Taiwan; yunhsuan@ems.cku.edu.tw; 6 Institute of Clinical Nursing, College of Nursing, National Yang Ming Chiao Tung University, Taipei 112, Taiwan; 7 Institute of Clinical Nursing, School of Nursing, National Yang-Ming University, Taipei 112, Taiwan

**Keywords:** clinical practice, menstrual distress, nursing students, Q-methodology, northern Taiwan

## Abstract

This study aimed to explore the cluster patterns of female nursing students’ perceptions of the effects of menstrual distress during clinical practice. This study adopted the Q-methodology study design. We recruited female nursing students from a college in northern Taiwan. Forty-seven Q-statements were constructed to explore participants’ experiences of the impact of menstrual distress on clinical learning. In total, 58 participants subjectively ranked Q-statements concerning menstrual distress experiences during clinical practice and were classified. After Q-sorting, the subjective ranking process PQ Method (version 2.35, Schmolck, Emmendingen, Germany) was employed for factor analysis. Four patterns of shared perspectives, accounting for 46.6% of the total variance, were identified: (a) influencing clinical learning and making good use of painkillers; (b) responsible attitudes and diversified relief of discomfort; (c) seeking peer support and effect on mood; (d) negative impact on learning ability and conservative self-care. Clinical practice is a major component of nursing education; menstrual distress affects female nursing students’ clinical learning and performance. The exploration of clustering different nursing students’ perceptions may facilitate customized strategies to enable more appropriate assistance.

## 1. Introduction

Menstrual distress affects female nursing students and influences their education and performance during clinical training. Menstrual distress is a common health problem for women; its symptoms include pain, water retention, autonomic reactions, negative affect, impaired concentration, arousal, loss of physical and psychological control, and behavioral changes [1]. A study focused on absenteeism during menstruation among Spanish nursing students revealed that the most common symptoms of menstrual discomfort in nursing students were bloating, dysmenorrhea, irritability, and fatigue [2]. A study on Turkish nursing students found that severe symptoms of menstrual discomfort resulted in a decrease in activities, an increase in tension, proneness to anger, hip and abdominal pain, backache, headache, and fatigue [3]. It even affects their alexithymia [4]. Nursing students believe that symptoms of menstruation cause not only physical discomfort and mood changes, but are also negative and limiting in that they make students feel less attractive [5].

These will have a negative impact on daily activities and school life. The most common effects are a lack of concentration in class, sleep disturbances, and course absenteeism [3]. Other studies have found similar results. Severe menstrual pain affects activities such as exercising, social interaction, health, class, personal studies, and academic performance [6]. It is worth noting that severe menstrual pain, among symptoms of menstrual distress, is more likely to cause absenteeism and negatively affect academic performance among nursing students [2,6,7]. Based on the aforementioned research results, we can conclude that several nursing students suffer from menstrual distress and that it may affect nursing students’ clinical learning and performance.

Clinical practice is a major component of the learning process in nursing education [8]. Clinical practice helps develop nursing students’ clinical performance and instill professional attitudes and behaviors, such as care, respect, accountability for patient care, prioritizing patients’ interests, honesty, and trustworthiness [9]. A sense of responsibility is critical in terms of nurses’ professional attitude because studies have shown a positive association between a sense of professional responsibility and the quality of patient care provided [10]. Clinical practice enables nursing students to apply nursing knowledge and skills in a real environment, observe and imitate the approaches of senior nurses, and adapt to their future professional roles [11]; clinical practice also enables students to adopt a professional appearance and develop appropriate communication skills [12]. This clinical education can improve the quality of care that nursing students provide to patients [13]. Thus, clinical education is very important for nursing students. However, clinical learning and providing care for patients can be stressful for nursing students [14]; additionally, stress has a notable impact on menstrual distress symptoms [15]. Therefore, the influence of menstrual distress on clinical practice is a topic that deserves thorough attention.

While facing the symptoms of menstrual distress, most nursing students manage menstrual distress primarily through conservative self-care; care strategies include rest, locally applying heat packs, drinking herbal tea, taking a hot shower, walking [3], antalgic positions, massages, and local heat [16]. Nursing students with moderate and severe menstrual pain reported using painkillers like non-steroidal anti-inflammatory drugs (NSAIDs); a majority of these nursing students choose to self-medicate [6,17,18].

Menstrual distress has different effects on different nursing students, and they resort to different coping strategies. When nursing students encounter clinical practice challenges, clinical instructors’ support is significant because the way they respond affects students’ clinical learning experience and attitude [14]. The clinical instructor should provide different strategies for different patterns of response employed by nursing students. Therefore, this study aimed to explore the cluster patterns of nursing students’ perceptions of the effects of menstrual distress during clinical practice. To achieve this, we applied the Q-methodology.

## 2. Materials and Methods

The Q-methodology combines the strengths of both qualitative and quantitative research [19]. In this approach, participants are presented with a set of statements regarding a designated topic and asked to rank these descriptive statements based on the degree to which they consider them relevant or correct. Individual perceptions are then obtained by examining respondents’ sorting of the set of statements.

### 2.1. Developing the Q-Sample

A Q-sample is a collection of statements about a phenomenon of interest. Q-statements were developed by inviting 10 nursing students with menstrual distress experiences to participate in face-to-face interviews and asked how these experiences impacted their clinical learning. Each interview, which lasted approximately 35–45 min, was audio-recorded, and subsequently transcribed verbatim. After the interviews, 177 statements were extracted from the verbatim transcripts, which were thoroughly reviewed by the research team and narrowed down to 52 statements. These interviews helped us to develop statements accurately, describing nursing students’ experiences and perceptions regarding menstrual distress. Initial categories were extracted from the transcripts. Five categories pertaining to their perceptions of menstrual distress during clinical practice were identified: Learning ability, clinical performance, sense of responsibility, interpersonal interaction, and discomfort relief. The content validity index (CVI) was used to evaluate the statements to form a Q-sample. Clinical nursing staff (*n* = 2) and nursing educators (*n* = 3) reviewed the statements for appropriateness and clarity, using a 4-point Likert scale. Lynn [20] suggested that a CVI ≥0.80 is acceptable; thus, only statements with CVI >0.80 were retained for further Q sorting. A modified list of 47 Q-statements was obtained, as per the experts’ feedback, by clarifying individual statements’ semantics and eliminating duplication. This was followed by a pre-test of the Q-sample with five nursing students to evaluate the comprehensibility and clarity of the statements. Finally, we used 47 Q-statements in this study (Table 1).

### 2.2. Obtaining the P-Set

The P-set represents the participant group. We recruited nursing students who had experienced menstrual distress during clinical practice, from a college in northern Taiwan. All participants had completed their 8-month clinical practice. A group of 40 to 60 participants is appropriate for a Q-methodology-based analysis [21]. Consequently, in this study, a total of 58 participants completed Q-ranking and were classified.

### 2.3. Administering the Q-Sort

**Figure 1:**
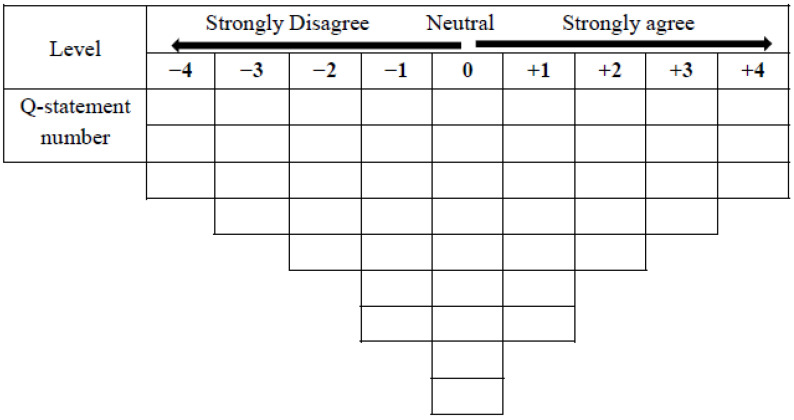
A Q-sort grid for rank-ordering the 47 Q-statements.

Q-sorting is a procedure that enables participants—nursing students in the present research—to rank Q-statements on a normally distributed 47-cell grid (Figure 1) in terms of the degree to which each statement represents their own experience. The participants were invited to read the cards comprising Q-statements. To familiarize participants with the cards and reduce the cognitive load, they were first asked to organize the cards into three piles: Q-statements they disagreed with, Q-statements regarding which they had no opinion, or Q-statements they agreed with. They then placed all statements on the grid, using the range of −4 (= strongly disagree) to 4 (= strongly agree) to determine the location of each statement. Data collection took approximately 35–45 min per participant.

### 2.4. Data Analysis

The exploratory factor analysis clustered participants who performed Q-sorts into groups, and the Q-sort data were entered into PQ Method version 2.35 [22]. The Q-factor analysis identified the factors and was performed using a principal components analysis with varimax rotation. This process produced a factor array and derived composite Q-sorts that represented the perception patterns associated with menstrual distress during clinical practice. The characterizing statements, ranked at the most positive ends of each factor’s composite Q-sort (i.e., +3, +4), were used to describe nursing students’ perceptions, who loaded significantly on to the four factors.

### 2.5. Ethical Considerations

All subjects provided informed consent before participating in the study. The study was conducted in accordance with the Declaration of Helsinki, and the protocol was approved by the Ethics Committee of the Mackay Memorial Hospital (No. 16MMHIS081e), on 22 November 2016. Participants were assured that their participation was voluntary, that they could refuse to participate or withdraw from the study without penalty, and that their participation would not affect their academic evaluations. All information collected was treated confidentially and anonymized. All participants provided written informed consent.

## 3. Results

A total of 58 nursing students were included, aged between 20 and 23 years (M_age_ = 20.1). Participants’ average menstrual pain score (0–10) on an index of experiencing dysmenorrhea was 5.48 points; the number of participants taking painkillers was 37 (63.8%); some participants took menstrual leave during clinical practice (*n* = 15, 25.9%). During the internship, 33 participants experienced an increase in menstrual discomfort (56.9%).

The authors then determined the number of retention factors by using a combination of feature values and screen plots. The Q-factor analysis revealed a four-factor solution that accounted for 46.6% of the total variance. The four factors’ explained variances were 23.2%, 9.6%, 7.5%, and 6.3%, respectively. The eigenvalues were 13.9, 5.7, 4.5, and 3.8, respectively.

Table 1 shows the corresponding categories, the 47 Q-statements, and factor arrays of the four factors. The Q-factor analysis generated clusters of nursing students with similar perceptions. The initial description is presented according to the characterizing statements ranked at the most positive ends (i.e., +3 and +4) of the composite Q-sort of each factor (Table 2). The characteristic statements of all the factors included the following two categories: “learning ability” and “discomfort relief”. The most special categories were the following: “clinical performance” (Factor I); “sense of responsibility” (Factor II); “interpersonal interaction” (Factor III); and “learning ability” (Factor IV). The research team implemented a consensus process to finalize explanations of the Q-sort. Four opinions were identified by nursing students who experienced menstrual distress during clinical practice (Table 2).

### 3.1. Factor I: Influencing Clinical Learning and Making Good Use of Painkillers

Five participants significantly identified with Factor I. Those participants were the only ones that emphasized nursing students’ clinical performance. Notably, clinical practice experience and performance were rated negatively: “I become impatient when listening to the demands of patients or their families” (+4); “I lose my enthusiasm to perform nursing tasks” (+4); and “I cannot care for my patients” (+3). For impact on learning ability, their perspectives included: “My problem-solving ability decreases” (+4); “My learning efficiency decreases” (+3); and “I cannot focus and am easily distracted” (+3). Following this, they deemed only one way to try and relieve discomfort: “I keep painkillers with me in case I need them” (+3) (Table 2). Compared with other participants, those associated with Factor I took the most painkillers (100%) (Table 3).

Overall, Factor I emphasized that students’ clinical performance and learning ability were negatively impacted; further, they make good use of painkillers.

### 3.2. Factor II: Responsible Attitudes and Diversified Relief of Discomfort

Twenty-one participants identified solely with Factor II. These participants were the only ones that emphasized nursing students’ sense of responsibility, although, 61.9% of the participants experienced increased menstrual distress during the internship (Table 3). Their perspectives on “sense of responsibility” included: “Remaining at my post, even when experiencing physical discomfort, is my responsibility” (+4); “I can endure menstrual distress because of my sense of responsibility” (+4); and “I cannot take time off to rest and recover because of my sense of responsibility” (+3). Compared with other participants, those associated with Factor II had the fewest number of people taking menstrual leave during clinical practice (14.3%) (Table 3). Additionally, they use diversified methods of relief to alleviate discomfort: “I mitigate my discomfort by applying a warm compress or resting” (+4); “I keep painkillers with me in case I need them” (+3); and “I practice self-care for menstrual distress” (+3). The related description that affects their learning ability is “I become easily fatigued” (+3) (Table 2).

Overall, Factor II showed that students endure menstrual distress because of their sense of responsibility, and they use diversified methods of relief to reduce discomfort.

### 3.3. Factor III: Seek Peer Support and Effect on Mood

Nineteen participants adhered solely to Factor III. Those participants were the only ones that emphasized interpersonal relationships. Their perspective was: “My classmates assist me when I ask for help” (+3). Mood changes significantly affect their learning ability, and their perspectives included: “I develop a bad mood and become prone to anger” (+4); “I cannot focus and am easily distracted” (+3); “The stress of learning increases my discomfort” (+3); and “I become easily fatigued” (+4). Compared with other participants, those associated with Factor III took the most time off/menstrual leave during clinical practice (42.1%), and 63.2% of the participants experienced increased menstrual distress during the internship (Table 3). They also try to relieve discomfort: “I mitigate my discomfort by applying a warm compress or resting” (+4); and “I practice self-care for menstrual distress” (+3) (Table 2).

Overall, students seek help from classmates, and their moods and learning ability are affected.

### 3.4. Factor IV: Negative Impact on Learning Ability and Conservative Self-Care

Thirteen participants adhered solely to Factor IV. The focus of this pattern of nursing students was on the impact of learning ability. Their perspectives included: “My problem-solving ability decreases” (+4); “The stress of learning increases my discomfort” (+4); “I become easily fatigued” (+4); “I cannot focus and am easily distracted” (+3); “My learning efficiency decreases” (+3); and “I develop a bad mood and become prone to anger” (+3). They try to relieve discomfort with conservative self-care: “I mitigate my discomfort by applying a warm compress or resting” (+3) (Table 2). Compared with other participants, the group associated with Factor IV took fewer painkillers (23.1%) (Table 3).

Overall, Factor IV indicated that nursing students’ learning ability was obviously negatively impacted and that they usually used conservative self-care practices to cope with their menstrual distress.

## 4. Discussion

This study explores experiences associated with the effects of menstrual distress during clinical practice with Q-factor analysis. It revealed four different perspectives among nursing students. The following describes the characteristics of each pattern one by one, and reminders were provided to the clinical instructor.

### 4.1. Factor I

The nursing students associated with Factor I emphasized that menstrual distress negatively impacted their clinical performance. Similarly, other studies have shown that menstrual distress decreased work and learning satisfaction levels of female students [6,23]. These results should not be ignored; it is necessary to pay attention to negative clinical performance. The categories relating to “impatient when listening to the demands of patients” and “cannot care for patients” may cause communication difficulties and affect quality of care. After all, a nursing profession is a form of altruism in which patients’ best interests are prioritized by providing quality care and service [11]. Besides, all nursing students associated with Factor I took and/or carried painkillers. The painkillers can reduce menstrual pain [6,17]; in addition to painkillers, clinical instructors are responsible for reminding students to practice self-care, including resting, locally applying heat packs, drinking herbal tea, and walking [3]. Through reminders, nursing students can implement appropriate self-care strategies. Students may also be reminded to reflect on whether menstruation distress harmed their clinical performance, and thereby avoid hindering the quality of care and clinical learning.

### 4.2. Factor II

The nursing students associated with Factor II emphasized responsible attitudes. Responsibility is part of the nursing profession [24] and central to nurses’ professional attitudes because studies have shown a positive association between professional responsibility and patient care quality [10]. In this group, 61.9% experienced increased menstrual distress during clinical practice, but they had the lowest menstrual leave rates. The reason for this is participating nursing students’ sense of responsibility; they believed that menstrual distress was not a reason to be absent [7]. For this reason, they endure discomfort and actively use different coping strategies. This learning ability of this approach pattern is the least affected. Related descriptions affecting learning ability are only “easily fatigued.” However, “fatigue” is a common symptom of nursing students’ menstrual discomfort and should not be ignored [2,3]. Clinical instructors should remind nurses to rest and seek support to avoid overburdening themselves. Whether their learning effect is the best is also worthy of follow-up research.

### 4.3. Factor III

Nursing students associated with Factor III were the only ones that emphasized interpersonal relationships; they can ask for help from classmates. Peer support during clinical practice is significant because learning partnerships can affect the quality of clinical performance [25]. Those associated with Factor III had the highest proportion of increased menstrual distress during clinical practice and took the most menstrual leaves during clinical practice. Similarly, previous studies have shown that menstrual discomfort can result in student absenteeism and adversely affect learning effectiveness [2,6,7]. It is worth examining whether these nursing students’ mood changes affect learning motivation. In addition, clinical practice experience and training time due to menstrual distress decreases, which affects their clinical learning outcomes. Clinical instructors need to be aware of the potential negative impacts of menstrual discomfort on student learning. Possible solutions include evaluating students’ learning progress and providing supplementary materials when needed.

### 4.4. Factor IV

The nursing students associated with Factor IV emphasized that menstrual distress negatively impacted their learning ability. Similarly, other studies have shown that menstrual distress resulted in decreased attention and poor learning outcomes for female students [6,23]. Compared with other participants, they took the least number of painkillers and few asked for menstrual leave. They adopt conservative self-care practices to cope with their menstrual distress. However, 46.2% experienced that the degree of menstrual distress increased during clinical practice. For students associated with this factor, clinical instructors should assess the extent of the effects of menstrual distress on clinical learning and their understanding of painkillers. Students should be reminded that there are more strategies to reduce the discomfort of menstrual distress.

Clinical instructors should evaluate whether menstrual distress affects the clinical learning effectiveness and comfort of nursing students. Nursing students experiencing menstrual distress, in addition to physical changes and mood swings, also seek a safe environment [5]. Clinical instructors should remind students to practice self-care and provide appropriate assistance. Through enhanced support, care, and a supportive environment, students can receive additional assistance and comfort during menstruation [26]. Future research could explore whether these four groups of nursing students following different patterns and approaches differ in their clinical learning effectiveness.

Based on our study findings, menstrual distress effects and the subsequent coping strategies vary for different nursing students. Previous studies have shown that menstrual discomfort can lead to student absenteeism and affect learning effectiveness adversely [2,6,7]. Furthermore, conservative self-care [3,16] and painkiller consumption [6,17,18] have been shown to facilitate menstrual distress management among nursing students. However, these studies are broad investigations, and they did not focus on the specific impact of menstrual distress during clinical practice; additionally, these effects and subsequent coping strategies were not classified in these studies. Therefore, the current study findings make novel contributions to the scholarship by elucidating the impact of menstrual distress, experienced by nursing students, during their clinical practice.

### 4.5. Limitations

This study’s primary limitation was that our participants were limited to a college in northern Taiwan; thus, the generalizability of the results and its extension to other locations may be limited. However, the Q-methodology is useful for clustering individuals of similar perspectives. Through this method, we can discover the various effects and patterns of menstrual distress experienced by diverse nursing students during their internships. This will allow clinical instructors to provide appropriate strategies and methods to improve the effectiveness of nursing education.

## 5. Conclusions

The novel aspect of this study lies in the fact that it focuses on the impact of menstrual distress experienced by nursing students of Taiwan during their clinical practice. The four perspectives identified can provide clinical instructors with information on developing appropriate strategies to reduce its negative impacts on clinical learning. The clinical learning achievement impact of these four patterns among nursing students deserves further exploration and research. Furthermore, nursing students should implement appropriate self-care strategies. These strategies may be important for achieving successful learning outcomes among this population.

## Figures and Tables

**Table 1:** The 47 Q-statements and the factor array scores of the four factors.

Categories—Q Statements	Factor Array Scores			
	F I	F II	F III	F IV
**Learning ability**				
- I cannot focus and am easily distracted.	3	0	3	3
- My problem-solving ability decreases.	4	0	0	4
- I tend to overlook details.	2	−1	0	1
- The stress of learning increases my discomfort.	2	1	3	4
- My learning efficiency decreases.	3	0	1	3
- I lack the motivation to think of solutions to problems.	0	−1	1	−1
- I become easily fatigued.	1	3	4	4
- I develop a bad mood and become prone to anger.	0	−2	4	3
**Clinical performance**				
- I cannot care for my patients.	3	−1	−3	−1
- I become impatient when listening to the demands of patients or their families.	4	−4	0	0
- I lose my enthusiasm to perform nursing tasks.	4	−3	−1	−2
- I lose my enthusiasm to engage in conversation.	0	0	2	2
- My impatience affects the nurse-patient relationship.	0	−4	0	−3
**Sense of responsibility**				
- Remaining at my post, even when experiencing physical discomfort, is my responsibility.	−1	4	−3	−1
- I can endure menstrual distress because of my sense of responsibility.	1	4	−1	2
- If I take leave, my classmates may lack understanding of my patients.	1	0	−1	0
- I cannot take time off to rest and recover because of my sense of responsibility.	1	3	0	−1
- Visibly showing pain or discomfort would undermine the nursing image.	2	2	−1	1
- I think that, in nursing, taking menstrual leave is not a good decision.	−2	−1	0	−2
- Other classmates do not take leave, so I do not dare to do so.	−1	−1	−3	−2
- I use willpower to endure the pain until the end of my clinical practice.	−2	2	2	2
- I feel guilty about the impatience I experience during clinical practice.	−4	−3	0	2
- Forcing myself to learn while experiencing discomfort makes me question my self-worth.	−4	−4	−3	−3
**Interpersonal interaction**				
- Taking menstrual leave creates difficulties for teachers.	2	2	−2	0
- Taking menstrual leave causes teachers to develop a negative impression of a student.	−1	1	−4	1
- Reducing my work efficiency may affect my relationship with my classmates.	0	0	−2	1
- I fear being labeled as having faked illness.	0	2	−2	0
- Taking menstrual leave would increase the workload for others.	1	2	−2	1
- My classmates find it difficult to learn with peers who are experiencing menstrual distress.	−1	−3	−4	0
- I cannot find classmates willing to substitute for me.	−2	−3	−2	−2
- In order to avoid owing favors to classmates, I prefer to endure the discomfort.	−3	−2	−4	−3
- Other classmates empathize with me and understand my situation.	−1	1	1	1
- I release my negative emotions away from my family.	−4	−2	0	1
- My classmates try to help when I suffer from pain and have difficulty moving.	−3	1	0	0
- The learning workplace is understanding of situations such as menstrual discomfort.	−3	−2	−1	−1
- My classmates assist me when I ask for help.	−2	1	3	0
- My classmates perform physical-labor-related tasks, allowing me to rest.	−3	−1	2	−1
**Discomfort relief**				
- I keep painkillers with me in case I need them.	3	3	2	−4
- I take non-prescription medicine to manage the discomfort.	1	0	1	−4
- I complete tasks swiftly so that I have time to sit down and rest.	0	1	1	0
- I mitigate my discomfort by applying a warm compress or resting.	−1	4	4	3
- I practice self-care for menstrual distress.	0	3	3	−1
- I take painkillers in advance to ease the pain and maintain my performance level.	2	1	1	−4
- I am concerned about developing a dependence on painkillers.	0	−1	−1	−2
- I take more painkillers if I anticipate a heavier workload.	1	−2	−1	−3
- Diverting my attention helps me to learn at a normal pace.	−1	0	1	2
- My only desire is to go home and lie down on the bed.	−3	0	2	0

The data listed in this table are sourced from the present study.

**Table 2:** Characterizing statements of the four factors.

Categories	Results of Q-Sort Factor Analysis			
	Factor I	Factor II	Factor III	Factor IV
Learning ability	My problem-solving ability decreases. (+4) I cannot focus and am easily distracted. (+3) My learning efficiency decreases. (+3)	I become easily fatigued. (+3)	I become easily fatigued. (+4) I develop a bad mood and become prone to anger. (+4) I cannot focus and am easily distracted. (+3) The stress of learning increases my discomfort. (+3)	My problem-solving ability decreases. (+4) The stress of learning increases my discomfort. (+4) I become easily fatigued. (+4) I cannot focus and am easily distracted. (+3) My learning efficiency decreases. (+3) I develop a bad mood and become prone to anger. (+3)
Clinical performance	I become impatient when listening to the demands of patients or their families. (+4) I lose my enthusiasm to perform nursing tasks. (+4) I cannot care for my patients. (+3)			
Sense of responsibility		Remaining at my post, even when experiencing physical discomfort, is my responsibility. (+4) I can endure menstrual distress because of my sense of responsibility. (+4) I cannot take time off to rest and recover because of my sense of responsibility. (+3)		
Interpersonal interaction			My classmates assist me when I ask for help. (+3)	
Discomfort relief	I keep painkillers with me in case I need them. (+3)	I mitigate my discomfort by applying a warm compress or resting. (+4) I keep painkillers with me in case I need them. (+3) I practice self-care for menstrual distress. (+3)	I mitigate my discomfort by applying a warm compress or resting. (+4) I practice self-care for menstrual distress. (+3)	I mitigate my discomfort by applying a warm compress or resting. (+3)

The data listed in this table are sourced from the present study. The numbers in the parentheses were identified by Q-sort factor analysis.

**Table 3:** Background information of the students within each of the four factors.

Variables	Factor I *n* = 5	Factor II *n* = 21	Factor III *n* = 19	Factor IV *n* = 13
Number of participants taking painkillers [*n* (%)]				
Yes	5 (100)	16 (76.2)	13 (68.4)	3 (23.1)
No	0	5 (23.8)	6 (31.6)	10 (76.9)
Experienced increased menstrual distress during clinical practice [*n* (%)]				
Yes	2 (40)	13 (61.9)	12 (63.2)	6 (46.2)
No	3 (60)	8 (38.1)	7 (36.8)	7 (53.8)
Took menstrual leave during clinical practice [*n* (%)]				
Yes	1 (20)	3 (14.3)	8 (42.1)	3 (23.1)
No	4 (80)	18 (85.7)	11 (57.9)	10 (76.9)

## Data Availability

The data presented in this study are available on request from the corresponding author.
